# An Update in Management of Noncutaneous T-Cell Lymphomas

**DOI:** 10.1155/2010/424786

**Published:** 2010-12-02

**Authors:** Y. Y. Hwang, R. H. S. Liang

**Affiliations:** Division of Haematology, Department of Medicine, Queen Mary Hospital, The University of Hong Kong, Pokfulam, Hong Kong

## Abstract

T-cell lymphoma is a heterogeneous group of diseases. Except for ALK positive anaplastic large cell lymphoma, T-cell lymphoma responds to conventional chemotherapy unfavourably, and most patients carry poor prognosis. In recent years, efforts have been made to improve the outcome of T-cell lymphoma patients. Novel agents, high-dose therapy, and allogeneic stem cell transplantation are studied, and various results are reported in literature. This paper looks into the prognostication and treatment approach of different entities of noncutaneous T-cell lymphoma and would focus on the latest updates in its management.

## 1. Introduction

T-cell lymphoma accounts for 10 to 15 percent of all non-Hodgkin lymphomas worldwide. In the latest WHO classification of Tumours of Haematopoietic and Lymphoid Tissues, there are altogether nineteen mature T-cell lymphoid malignancies [1]. All of them carry poor prognosis with the notable exception of anaplastic lymphoma kinase (ALK)- positive anaplastic large cell lymphoma. They in general respond poorly to conventional chemotherapy with the reported 5-year overall survival (OS) rate and event-free survival (EFS) rate of 41% and 33%, respectively [2], which compares unfavourably with B-cell lymphoid neoplasms [2–4]. 

International Prognostic Index (IPI) was originally developed to prognosticate B-cell lymphoma patients. It has been used in T-cell lymphoma patients, but its applicability in them has been doubted [5]. Definitely, there is a need of a reliable prognostic model to stratify this group of patients.

Various attempts were made to improve the dismal outcome of T-cell lymphomas. These include different combinations of conventional chemotherapy, novel agents, high-dose therapy, and allogeneic stem cell transplantation. Despite all these advances, cyclophosphamide, doxorubicin, vincristine, and prednisolone- (CHOP-) like therapy are still the standard first-line treatment.

This paper looks into the various prognostic markers in T-cell lymphoma as well as the latest updates in its management.

## 2. T-Cell Lymphomas: Clinical Characteristics

Among the nineteen subtypes of T-cell lymphomas, angioimmunoblastic T-cell lymphoma, anaplastic large cell lymphoma, and peripheral T-cell lymphoma not otherwise specified (PTCL-NOS) are the most commonly seen. They each accounts for 20% to 30% of all T-cell lymphomas [1]. The median age at presentation is between the fifth and sixth decade with a male predominance, with the exception of ALK-positive anaplastic large cell lymphoma which is usually diagnosed in patients younger than 30 [1, 6].

All T-cell lymphomas behave aggressively except ALK-positive anaplastic large cell lymphoma. They present at an advanced stage, and almost all patients have high IPI scores. A great majority of them had bone marrow and extranodal involvement upon diagnosis. The reported 5-year overall survival and progression-free survival ranged between 32% to 49% and 18% to 36%, respectively [7]. It is important to distinguish cases of ALK-positive anaplastic large cell lymphoma in analysis of T-cell lymphomas because of its exceptional good prognosis. The 5-year overall survival of this group of patients reaches 80% [8, 9].

## 3. Prognostic Markers in T-Cell Lymphoma

IPI has been the standard prognostic model for B-cell lymphomas for years. It has been applied in T-cell counterparts as well. It stratified anaplastic large cell lymphomas (both ALK positive and ALK negative) and PTCL-NOS into two prognostic groups. The overall survival at 5 years is significantly better in patients with IPI scores less than 1 than those with scores higher than 4 (50% to 90% versus 11% to 33%) in these two types of T-cell lymphomas [7]. However, IPI cannot differentiate high-risk patients in some rare subtypes of T-cell lymphomas like enteropathy-associated T-cell lymphoma, hepatosplenic T-cell lymphoma, and adult T-cell leukemia/lymphoma. Patients with these lymphomas fared poorly even with a low IPI score. IPI also has limited applicability in angioimmunoblastic T-cell lymphoma because most patients have high IPI scores at presentation [5, 10]. A prognostic model was specifically developed for PTCL-NOS, the Prognostic Index for PTCL-U, (PIT). It consists of age, performance status, lactate dehydrogenase level, and bone marrow involvement and divides patients into four groups depending on the number of adverse factors they have [11]. It showed a superior predictive power of survival when compared with IPI in this study (log-rank 66.79 versus 55.94). However, even for patients with no adverse factor (i.e., group 1), the 5-year overall survival rate was only 62%. Therefore, it is apparent that a great majority of T-cell lymphoma patients, with the exception of ALK-positive anaplastic large cell lymphoma, have a dismal prognosis irrespective of their prognostic scores.

## 4. Advances in Management of T-Cell Lymphomas

Conventional CHOP-like chemotherapy yields poor response in T-cell lymphomas excluding ALK-positive anaplastic large cell lymphoma. The reported complete remission rate with CHOP-like regimens was less than 50%, and the 5-year overall survival ranged from 26% to 45% [4, 12, 13]. This result is certainly inferior to B-cell lymphomas even prior to the era of Rituximab. The use of anthracycline does not bring any improvement in outcome either [4, 7]. A recent trial reported that the addition of etoposide to CHOP improved event-free survival in young patients with ALK-positive anaplastic large cell lymphomas (event-free survival at 3 years 91.2% versus 57.1%, *P* = .012), but the overall survival was not significantly different. There was a trend towards a better event-free survival in the remaining T-cell lymphoma patients, but it did not reach statistical significance (3-year event-free survival 60.7% versus 48.3%, *P* = .057) [14]. 

## 5. Intensive Chemotherapy Regimens

Intensive chemotherapy did not produce improvement in treatment results in this group of patients. GELA (Groupe d'Etudes des Lymphomes) showed that intensive ACVBP regimen (doxorubicin, cyclophosphamide, vindesine, bleomycin, and prednisolone) was better than CHOP in terms of overall survival and event-free survival (5-year overall survival rates 46% versus 38% (*P* = .036) and 5-year event-free survival 39% versus 29% (*P* = .007)). However, only 15% of the total 635 patients had T-cell lymphomas, and the outcome of this subgroup of patients was not reported separately [16]. In studies that only included T-cell lymphoma patients, none of them showed that dose-intense therapy improved outcome. In fact, a subsequent report by GELA group showed that ACVBP was not superior to conventional CHOP in treatment of angioimmunoblastic T-cell lymphoma patients [5]. MD Anderson group compared CHOP with intensive regimens (hyper-CHOP, hyper-CVAD, and alternating triple therapy) in 135 T-cell lymphoma patients (including 40 anaplastic large cell lymphoma patients). There was no significant difference in overall survival as well as complete remission rates between the two groups. Similar results were obtained when the patients with anaplastic large cell lymphoma were excluded from analysis [15].  Table 1 summarized the results of these studies. In conclusion, there was so far no regimen shown to be superior to CHOP in the literature.

## 6. High-Dose Therapy and Autologous Stem Cell Transplantation

Studies evaluating the role of autologous stem cell transplantation in treatment of T-cell lymphomas showed conflicting results. Autologous stem cell transplantation failed to improve survival for angioimmunoblastic T-cell lymphoma patients in a GELA study. However, the disease status of patients at transplantation was not known [5]. The same group reported another study comparing the outcome of aggressive lymphomas with either autologous stem cell transplantation or chemotherapy consolidation. They received induction chemotherapy followed by consolidation with either autologous stem cell transplantation or chemotherapy if they achieved complete remission. They did not find any significant difference in remission rates and survival in the subgroup of patients with nonanaplastic T-cell lymphoma [17]. A recent study in Germany also found that frontline high-dose therapy (Mega CHOP plus etoposide) with autologous stem cell transplantation had complete remission rate of less than 50% in patients with nonanaplastic T-cell lymphomas [18]. The overall survival and event-free survival at 3 years were 25.9% (95% C.I. 10.4%–41.4%) and 44.5% (95% C.I. 26.5%–62.5%), respectively, which was significantly worse than B-cell counterparts. Moreover, in 33 T-cell lymphoma patients enrolled in this study, only 22 patients (66.7%) were able to undergo autologous stem cell transplantation as per protocol. The majority of early drop-outs was due to disease progression during therapy. 

On the contrary, some better results were reported when autologous stem cell transplantation was done in first complete remission. A retrospective study in Spain showed the 5-year overall survival rate and progression-free survival rate of 68% and 63%, respectively, when autologous stem cell transplantation was done in first complete remission [19]. However, 31% of the patients were anaplastic large cell lymphomas, and the status of ALK expression was not known. Even when anaplastic large cell lymphomas were excluded from analysis, the overall survival and progression-free survival rate at 5 years were 61% and 55%, respectively, which is remarkably better than the results obtained with conventional chemotherapy. The same group reported a superior outcome with autologous stem cell transplantation as consolidation for patients with peripheral T-cell lymphoma (excluding cases with ALK-positive anaplastic large cell lymphoma), who achieved a complete or partial remission after induction chemotherapy in a prospective trial [20]. The complete remission rate was 89% after autologous stem cell transplantation, and the overall survival and progression-free survival were 73% and 53%, respectively, at 3 years. In other prospective trials of 83 patients with T-cell lymphomas, autologous stem cell transplantation was done in 55 of enrolled patients who achieved at least a partial remission after induction chemotherapy. The complete remission rate after stem cell transplantation was 78%, and the overall survival at 3 years was 71% [21]. In two prospective studies done by an Italian group, 46 (74%) of 62 enrolled patients underwent autologous stem cell transplantation as consolidation after they achieved complete remission with induction chemotherapy. After a median followup of 76 months, the estimated overall survival and event-free survival at 12 years were 34% and 30%, respectively. When patients with ALK-positive anaplastic large cell lymphomas are excluded, the overall and event-free survival at 12 years dropped to 21% and 18%, respectively [22].

Based on these retrospective and prospective studies, it seems that autologous stem cell transplantation is beneficial if it is done in first complete remission and when the disease is chemosensitive. Unfortunately, it is apparent that a significant number of patients have progression early in disease course such that autologous stem cell transplantation is not feasible at all. It is therefore the challenge how to improve the complete remission rate in nonanaplastic T-cell lymphomas and how to salvage patients who do not respond to frontline induction chemotherapy.

## 7. Novel Agents

Alemtuzumab is a humanized monoclonal anti-CD 52 antibody. CD 52 is expressed by both T-cells and B-cells. It was shown to produce superior remission rate (complete remission of 71%) when alemtuzumab was combined with CHOP as frontline treatment in a group of 24 patients with peripheral T-cell lymphomas (none of the them were ALK-positive anaplastic large cell lymphoma) [23]. The median duration of complete remission was 11 months. However, there was a high incidence of infectious complications despite anti-infective prophylaxis. The marked immunosuppression associated with alemtuzumab is a major issue that needs to be addressed when this antibody is administered.

Gemcitabine, a pyrimidine analogue, was studied in peripheral T-cell lymphomas either as single agent [24, 25] or combination treatment [26, 27] in both frontline and salvage setting. An overall response rate from 51% to 77% was achieved. It was well tolerated with mainly haematological toxicity reported. However, only a small number of patients were enrolled in each of these studies, and well-designed randomized controlled trials are needed before its role is defined in T-cell lymphoma management.

Pralatrexate is a new folate antagonist which is structurally similar to methotrexte but with greater affinity for one carbon-reduced folate carrier, and hence it is selectively accumulated in malignant cells. Both in vitro and in vivo data demonstrated superior efficacy of pralatrexate compared with methotrexate [28, 29]. It was demonstrated to have activity in peripheral T-cell lymphomas with an overall response rate of 54% achieved in a phase II study [30]. In the largest prospective study of relapsed or refractory peripheral T-cell lymphomas, Pralatrexate in Relapsed or Refractory Peripheral T-cell Lymphoma (PROPEL), an overall response rate of 29% was seen in 109 heavily pretreated patients, and the median duration of response was 9.4 months [31]. The main toxicity reported was mucositis and cytopenia, both of which were manageable. This result is impressive and led to its approval by Food and Drug Administration for treatment of relapsed or refractory peripheral T-cell lymphoma.

Histone deacetylase inhibitors are also shown to have activities in T-cell lymphomas. Vorinostat and romidepsin are approved by FDA for treatment of cutaneous T-cell lymphoma. A phase II study of romidepsin in 48 relapsed or refractory peripheral T-cell lymphoma patients found an overall response rate of 31% and a median duration of response of 9 months [32]. Ongoing studies are underway to evaluate the activity of this group of agents in peripheral T-cell lymphomas.

Apart from the above agents, there are some early results of novel agents such as mTOR (mammalian target of rapamycin) inhibitors [33], monoclonal antibodies anti-CD30 [34], and anti-CD4 [35] that they may be active in T-cell lymphomas. In addition, proteosome inhibitor bortezomib [36], purine analogue clofarabine [37], and lenalidomide [38] are also reported to be useful in T-cell lymphomas. However, more clinical trials on them are necessary before their efficacy in T-cell lymphoma management is determined.

## 8. Allogeneic Stem Cell Transplantation

There are encouraging results of allogeneic stem cell transplantation in management of peripheral T-cell lymphoma. European Group for Blood and Marrow Transplantation reported a retrospective study of 45 angioimmunoblastic T-cell lymphoma patients who received at least two lines of chemotherapy prior to study entry. They showed that the 3-year overall survival rate and progression-free survival rate of these heavily pretreated patients were 64% and 54%, respectively [39]. The cumulative incidence of relapse at 3 years was only 20%, and the cumulative incidence of nonrelapse mortality at 12 months after transplantation was 25%. The development of chronic graft-versus-host disease was associated with a lower incidence of relapse, but the difference did not reach statistical significance. Patients with chemosensitive diseases had a significantly better outcome (overall survival at 3 years 81% versus 64%,  *P* = .002; progression-free survival at 3 years 66% versus 53%,  *P* = .004). Another study from France which included 77 T-cell lymphoma patients undergoing allogeneic stem cell transplantation showed that the 5-year overall survival and event-free survival rate were 57% and 53%, respectively [40]. Better overall and event-free survival at 5 years was observed in those transplanted in complete or partial remission than patients with chemoresistant diseases (5-year overall survival 69% versus 29%; 5-year event-free survival 64% versus 27%,  *P* = .0002). Two patients in this study relapsed and achieved a second complete remission after donor lymphocyte infusion. Both of them had remained in remission for at least two years after donor lymphocyte infusion. In addition, 26% of patients in this study underwent reduced intensity conditioning (RIC) allogeneic stem cell transplantation, and they showed a trend of longer event-free survival and less transplant-related mortality in univariate analysis (*P* = .108 and  .107 resp.). This suggested a significant role of graft-versus-lymphoma effect in T-cell lymphomas. In fact, RIC allogeneic stem cell transplantation is an attractive option, particularly in patients who are heavily pretreated, aged, or with comorbidities. Some favourable results of RIC allogeneic stem cell transplantation in T-cell lymphomas are reported. In a prospective trial in Italy, 17 patients with peripheral T-cell lymphomas underwent RIC allogeneic transplantation. The overall survival and progression-free survival at 3 years were 81% and 64%, respectively, while the nonrelapse mortality was only 6% at 2 years [41]. Another recently published study done in United States showed a slightly inferior outcome with the overall survival and progression-free survival at 3 years of 59% and 53%, respectively, while the nonrelapse mortality at 3 years was 19% [42]. However, the median age of patients enrolled in this study was 57 (compared with median age of 41 in the former study), and a larger proportion of patients had chemoresistant disease at transplantation. Notwithstanding, based on the above results, RIC allogeneic transplantation appears to be a feasible and effective treatment option with acceptable toxicity for T-cell lymphomas.

## 9. Conclusion

T-cell lymphomas are a heterogeneous group of diseases but are uniformly aggressive and respond poorly to our conventional chemotherapy, with the notable exception of ALK-positive anaplastic large cell lymphoma. Some advances were made in its treatment in recent years, and some novel agents show early promising results.

Allogeneic stem cell transplantation should be considered in patients with poor-risk disease, and RIC extended this option to the older, more heavily pretreated patients. It is hoped that with the availability of novel agents, we are able to bring more patients into complete remission before transplantation and improve their outcome.

## Figures and Tables

**Table 1:** Summary of various treatment results of T-cell lymphomas.

Author	Year	No. of patients	ALK + ve ALCL	Regimens	CR	ORR	OS	EFS	Ref
Lopez-Guillermo et al.	1998	174	30 (ALK status not known)	120 CHOP 54 not specified	49%	64%	38% (at 4 years)	Not available	[4]
Armitage et al.	1989	134	Not known	80 CHOP-like 54 not specified	50%	Not available	45% (at 4 years)	Not available	[12]
Rudiger et al.	2002	129	0	90 CHOP-like 39 not specified	Not available	Not available	26% (at 5 years)	20% (at 5 years)	[13]
Schmitz et al.	2010	343	78	122 CHOP 221 CHOEP	Not available	Not available	53.9%–67.5% (at 3 years)	41.1%–50.0% (at 3 years)	[14]
Mourad et al.	2008	157	0	ACVBP + CHOP-like regimens	46%	Not available	33% (at 5 years)	29% (at 5 years)	[5]
Escalon et al.	2005	24	0	CHOP	58%	Not available	43% (at 3 years)	Not available	[15]
Escalon et al.	2005	52	0	HyperCVAD like	59%	Not available	49% (at 3 years)	Not available	[15]
